# Factors associated with an unfavourable outcome in elderly intensive care traumatic brain injury patients. a retrospective multicentre study

**DOI:** 10.1186/s12877-022-03651-x

**Published:** 2022-12-30

**Authors:** Y Launey, A Coquet, S Lasocki, C Dahyot-Fizelier, O Huet, E Le Pabic, A Roquilly, P Seguin

**Affiliations:** 1 Service de Réanimation Chirurgicale. CHU de Rennes. Hôpital Pontchaillou. 2, Rue Henri Le Guilloux, 35033 Rennes Cedex, France; 2 Département d’Anesthésie Réanimation, CHU de Angers, Angers, France; 3 Département d’Anesthésie Réanimation, CHU de Poitiers, Poitiers, France; 4 Département d’Anesthésie Réanimation, CHU de Brest, Brest, France; 5 Centre d’Investigation Clinique, CHU de Rennes, 2 Rue Henri Le Guilloux, 35000 Rennes, France; 6 Département d’Anesthésie Réanimation, CHU de Nantes, Nantes, France

**Keywords:** Outcome, Traumatic brain injury, Elderly, Intensive care unit

## Abstract

**Background:**

Changes in the epidemiology of traumatic brain injury (TBI) in older patients have received attention, but limited data are available on the outcome of these patients after admission to intensive care units (ICUs). The aim of this study was to evaluate the outcomes of patients over 65 years of age who were admitted to an ICU for TBI.

**Methods:**

This was a multicentre, retrospective, observational study conducted from January 2013 to February 2019 in the surgical ICUs of 5 level 1 trauma centres in France. Patients aged ≥ 65 years who were hospitalized in the ICU for TBI with or without extracranial injuries were included. The main objective was to determine the risk factors for unfavourable neurological outcome at 3 months defined as an Extended Glasgow Outcome Scale (GOSE) score < 5.

**Results:**

Among the 349 intensive care patients analysed, the GOSE score at 3 months was ≤ 4 and ≥ 5 in 233 (67%) and 116 (33%) patients, respectively. The mortality rate at 3 months was 157/233 (67%), and only 7 patients (2%) fully recovered or had minor symptoms. Withdrawal or withholding of life-sustaining therapies in the ICU was identified in 140 patients (40.1%). Multivariate analysis showed that age (OR 1.09, CI 95% 1.04–1.14), male sex (OR 2.94, CI95% 1.70–5.11), baseline Glasgow Coma Scale score (OR 1.20, CI95% 1.13–1.29), injury severity score (ISS; OR 1.04, CI95% 1.02–1.06) and use of osmotherapy (OR 2.42, CI95% 1.26–4.65) were associated with unfavourable outcomes (AUC = 0.79, CI 95% [0.74–0.84]). According to multivariate analysis, the variables providing the best sensitivity and specificity were age ≥ 77 years, Glasgow Coma Scale score ≤ 9 and ISS ≥ 25 (AUC = 0.79, CI 95% [0.74–0.84]).

**Conclusions:**

Among intensive care patients aged ≥ 65 years suffering from TBI, age (≥ 77 years), male sex, baseline Glasgow coma scale score (≤ 9), ISS (≥ 25) and use of osmotherapy were predictors of unfavourable neurological outcome.

**Trial registration:**

ClinicalTrials.gov Identifier: NCT04651803. Registered 03/12/2020. Retrospectively registered.

## Introduction

Traumatic brain injury (TBI) accounts for approximately 37% of all injury-related deaths in Europe, and particularly for patients ≥ 65 years old and in the most severe case (Glasgow coma score ≤ 8), with mortality rates between 31 and 51% [1, 2]. In high-income countries, TBI is increasing in the elderly population, with falls currently the leading cause [2]. In parallel, in 2019, it was reported 703 (9%) million persons aged ≥ 65 years in the global population, and this proportion is projected to rise further to 16% in 2050 [3]. Accordingly, we could expect that TBI in elderly individuals would be increasing, as recently reported between 2007 and 2016 [4], and this may explain why mortality has not improved in recent decades [2].

From 1997 to 2007, in three neurosurgical intensive care units (ICUs), the 6-month mortality rate in patients aged 70–79 and ≥ 80 years was 59% and 79%, respectively [5]. In severe elderly (≥ 65 years) TBI patients admitted to the ICU, the hospital and 6-month mortality rates were 64.6% and 72.9%, respectively [4]. Furthermore, elderly patient have a higher risk of long term disability due to comorbidities, frequent use of oral anticoagulants and/or antiplatelet medications, and/or prior brain disorders [2, 6, 7]. In TBI patients, age (> 59 years) was reported as the main factor linked to a poor outcome in ICU [5]. In addition, despite less severe TBI, elderly TBI patients (≥ 65 years) had a worse functional outcome and higher mortality than younger patients [8]. Since there is no consensus guideline for triage, identifying elderly patients who might benefit from ICUs remains challenging [9]. Traumatic brain-injured patients are particularly concerned about this issue. Indeed, some elderly TBI patients may recover well despite severe TBI and early aggressive management, and *a contrario* may experience unfavourable outcomes despite moderate TBI [10, 11]. Nevertheless, few data are available related to risk factors for unfavourable outcomes in elderly TBI patients requiring ICU admission [4, 5].

Our study's purpose was to determine the factors associated with unfavourable functional outcomes at 3 months in patients aged ≥ 65 years who were hospitalized in the ICU and suffering from TBI.

## Patients and methods

### Study setting and design

We conducted a multicentre, retrospective, observational study from January 2013 to February 2019 in the surgical ICUs of 5 level 1 trauma centres in France. Each centre is a referral trauma-ICU for their region, involved in the care of over 50 TBI patients per year. We collected data from the AtlanRéa database. The AtlanRéa database prospectively and consecutively registers numerous pieces of information about brain injury and/or trauma patients hospitalized in ICUs. Patients who died within 24 h of hospitalization, were resuscitated and awaited organ donation and/or refused study participation according to the patient or relatives were not included in the database. Data were collected by clinical research assistants in each participating ICU using an electronic case report form. We assessed data regarding outcomes after discharge from ICUs through phone interviews led by dedicated clinical research assistants. The AtlanRéa database was approved by the French data protection authority (Commission Nationale de l'Informatique et des Libertés, authorization no. 912662), and the present work was approved by the ethical committee of Rennes, France, according to French legislation (Avis n°20.145, date of acceptance: 23/11/2020) [12]. ClinicalTrials.gov Identifier: NCT04651803. Registered 03/12/2020. Retrospectively registered.

We included patients aged ≥ 65 years who were hospitalized in the ICU for TBI with or without extracranial injuries. Patients who were admitted for another cause of brain injury and those with missing data related to the main objective of the study (outcome at 3 months) were excluded from the analysis.

The main objective was to determine the factors associated with unfavourable neurological outcomes at 3 months in patients aged ≥ 65 years, suffering from TBI and hospitalized in the ICU.

### Data collection

Age, sex and body mass index were recorded for each patient. Previous chronic obstructive pulmonary disease (Global Initiative for Chronic Lung Disease < 80%), cardiac insufficiency, chronic renal failure defined as an estimated glomerular filtration rate less than 60 ml/min.1.73 m^−1^, diabetes, neoplastic history, stroke, active smoking and chronic alcoholism were recorded. The mechanism of injury (road traffic accidents, low falls [from standing] or high falls and other types of injury) was specified. The Glasgow Coma Scale (GCS) score was determined in the prehospital setting or at admission to the hospital before intubation and/or sedation, and the presence of at least one nonreactive and dilated pupil at the initial management was collected. Severe TBI was defined as a GCS score ≤ 8. The initial CT scan was classified according to the Marshall classification into six categories (diffuse injury I, II, III, IV, evacuated mass lesion (V) and no evacuated mass lesion (VI)) [13]. We assessed the severity of illness according to the Injury Severity Score (ISS), the Simplified Acute Physiology Score II (SAPS II), the SAPS II without age, and the Sequential Organ Failure Assessment (SOFA) score [14–16]. We also specified the severity by the Abbreviated Injury Scale (AIS) with the corresponding AIS score and the cumulative number of injuries [17]. Isolated TBI was considered TBI regardless of the AIS score, with a maximum AIS score of 1 for other injuries.

During each patient’s hospitalization, we collected the following data: overall use of intracranial pressure monitoring and specifically in the most severe patients (GCS score ≤ 8), occurrence of intracranial hypertension (defined as an intracranial pressure > 20 mmHg at least 15 min), and use of osmotherapy and/or barbiturates and/or hyperventilation. Neurosurgical procedures included extra or subdural haematoma, lobectomy and/or decompressive craniectomy. The occurrence of infections, acute respiratory distress syndrome (ARDS) and renal replacement therapy due to acute renal failure were recorded. We specified the need for mechanical ventilation, tracheostomy and the administration of vasopressors and the duration of mechanical ventilation and vasopressors.

We recorded the length of stay in the ICU and 3-month mortality rate. Patient outcomes were assessed using the Glasgow Outcome Scale Extended (GOSE) score at 90 days [18]. We dichotomized the GOSE score between the four lower values (corresponding to unfavourable outcome—GOS score 1 to 4) and the four upper values (corresponding to favourable outcome – GOSE score 5 to 8). We reported the details for each category of the GOSE. We also recorded the decision in the ICU to withdraw or withhold life support.

### Statistical analysis

We performed all statistical analyses using SAS v9.4 (SAS Institute Inc., Cary, NC, USA). Quantitative continuous and qualitative variables are expressed as the median (interquartile range) and number (percentage). We compared categorical variables and continuous variables using the Chi^2^ or Fisher exact tests, as appropriate, and Wilcoxon tests, respectively. Logistic univariate regressions were used to define the variables associated with unfavourable outcome (GOSE < 5) score at 3 months. The variables with a *p* value < 0.20 were included in a multivariate model, and a backwards stepwise procedure was used. The odds ratios (ORs) and 95% confidence intervals (95% Cis) were calculated. To evaluate the goodness-of-fit of the model, the Hosmer–Lemeshow test was used. Discrimination was assessed with the area under the curve (AUC). In the case of quantitative(s) variable(s) independently associated with unfavourable outcomes, receiver operating characteristic (ROC) curves were drawn by plotting the sensitivity *vs*. one minus the specificity, and the optimal threshold was determined as the threshold that maximizes sensitivity + specificity-1 (Youden Index). We considered a *p* value < 0.05 statistically significant for all comparisons.

## Results

**Fig. 1:**
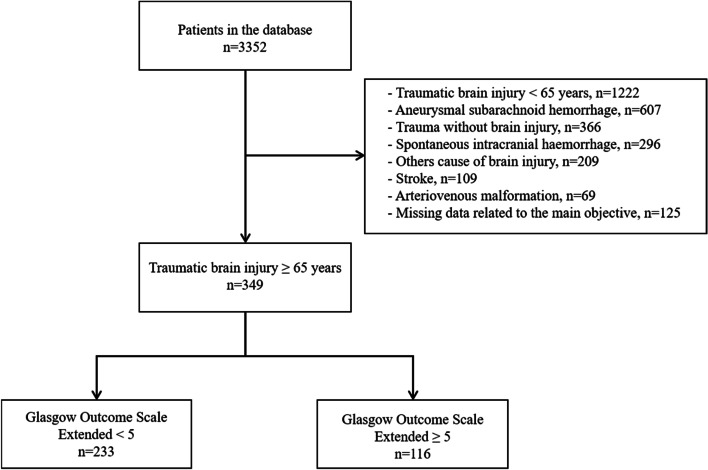
Flow chart

During the study period, 3352 patients were included in the database, and 349 patients ≥ 65 years old were analysed (Fig. 1).

**Fig. 2:**
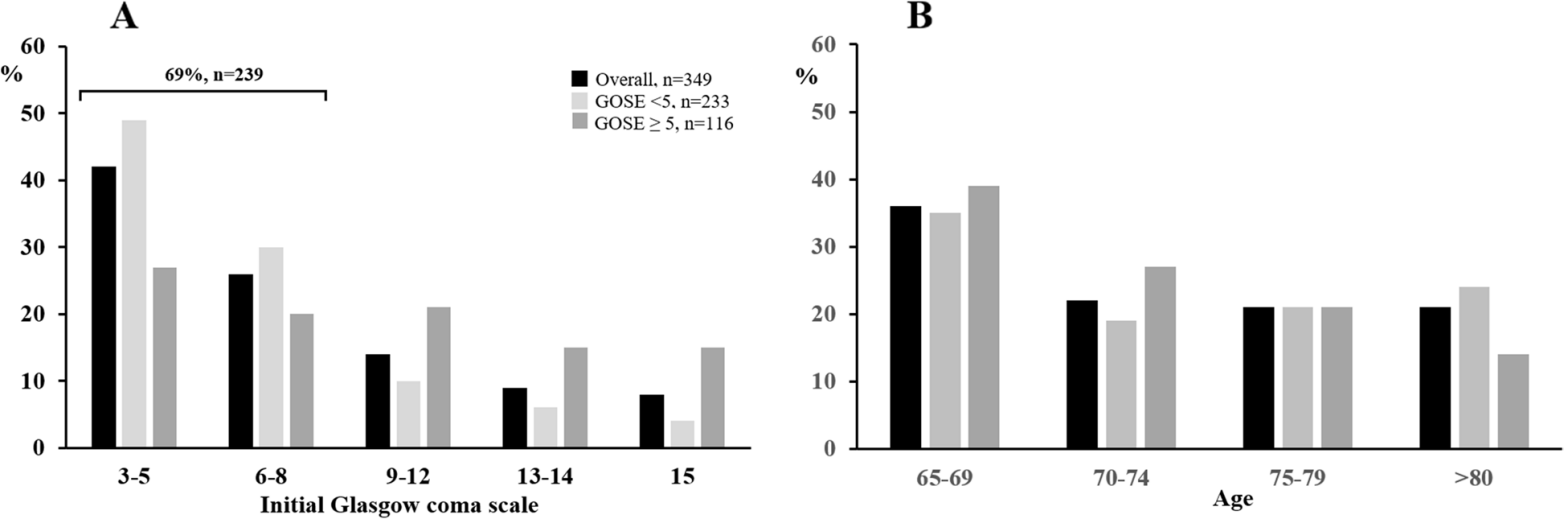
Glasgow Outcome Scale Extended score according to the baseline Glasgow Coma Scale score (**A**) and age (**B**)

Patients were predominantly male with a median (IQR [range]) age of 72 years (68–79 [65–95]) (Table 1). The median (IQR [range]) GCS score at baseline was 6 (3–10 [3–15]), and 239 patients (69%) had a GCS score ≤ 8 (Table 1 – Fig. 2). The TBI was isolated in 162 (46.4%) patients (Table 1).

**Table 1:** Baseline characteristics

		** ≥ 65 years**				
**Variables**	**Total (*n* = 349)**	**GOSE score ≤ 4 (*n* = 233)**	**Number of patients with data**	**GOSE score ≥ 5 (*n* = 116)**	**Number of patients with data**	**p**
Age, years	72 [68–79]	73 [68–79]	233	72 [67–77]	116	0.036
Males/females, ratio	2.0	2.5	233	1.3	116	0.004
Body mass index	26 [23–29]	26 [24–29]	209	26 [23–29]	109	0.896
Pre-existing conditions			233		116	
- Respiratory disease	21 (6.0)	18 (7.7)		3 (2.6)		0.057
- Cardiac insufficiency	17 (4.9)	11 (4.7)		6 (5.2)		0.854
- Chronic renal disease	13 (3.7)	9 (3.9)		4 (3.4)		1.000
- Diabetes mellitus	58 (16.6)	37 (15.9)		21 (18.1)		0.599
- Malignancy	22 (6.3)	16 (6.9)		6 (5.2)		0.540
- Stroke	28 (8.0)	18 (7.7)		10 (8.6)		0.772
- Alcohol addiction	52 (14.9)	36 (15.5)		16 (13.8)		0.682
- Active smoking	27 (7.7)	18 (7.7)		9 (7.8)		0.991
Mechanisms of injury			200		100	0.545
Road accident	97 (32.3)	61 (30.5)		36 (36.0)		
Low fall	110 (36.7)	73 (36.5)		37 (37.0)		
High fall	59 (19.7)	40 (20.0)		19 (19.0)		
Other mechanisms	34 (11.3)	26 (13.0)		8 (8.0)		
Glasgow Coma Scale score	6 [3–10]	6 [3–8]	233	10 [5–14]	116	< 0.001
Severe traumatic brain injury (GCS score ≤ 8)	239 (68.5)	184 (79.0)	233	55 (47.4)	116	< 0.001
Isolated traumatic brain injury*	162 (46.4)	108 (46.4)	233	54 (46.5)	116	1.00
Pupils (one or two dilated and nonreactive),	89 (26)	69 (30)	229	20 (17)	115	0.011
Marshall CT classification			200		85	0.019
I: Diffuse injury, no visible intracranial pathology	9 (3.2)	2 (1.0)		7 (8.2)		
II: Diffuse injury, midline shift of 0 to 5 mm	70 (24.6)	45 (22.5)		25 (29.4)		
III: Diffuse injury, basal cisterns compressed/effaced	12 (4.2)	10 (5.0)		2 (2.4)		
IV: Diffuse injury, midline shift > 5 mm	10 (3.5)	8 (4.0)		2 (2.4)		
V: Evacuated mass lesion	82 (28.8)	57 (28.5)		25 (29.4)		
VI: No evacuated mass lesions	102 (35.8)	78 (39.0)		24 (28.2)		
Injury Severity Score	25 [16–34]	25 [17–36]	233	20 [12–29]	116	< 0.001
Severity Acute Physiologic Score II	55 [44–65]	57 [46–67]	231	49 [41–60]	114	< 0.001
Severity Acute Physiologic Score II without age	40 [30–50]	43 [31–52]	231	35 [26–45]	114	< 0.001
Sequential Organ Failure Assessment score	8 [5–10]	9 [6–11]	233	7 [4–10]	116	< 0.001

Data are expressed as the median (25-75^th^ percentile) and number (percentage). GOSE: Glasgow Outcome Scale Extended. *Isolated traumatic brain injury was defined as TBI regardless of the AIS score, with a maximum AIS score of 1 for other injuries. ** Traumatic brain injury regardless of the AIS score for other injuries

The locations of body injuries, the AIS for each injury site and the cumulative number of injuries are provided in Table 2. Neurosurgical intervention was required in 92 (26%) patients (Table 3). Withdrawal or withholding of life-sustaining therapies in the ICU was applied in 140 (40.1%).

**Table 2:** Locations of bodily injuries, abbreviated injury scale and cumulative number of injuries

		** ≥ 65 years**				
**Variables**	**Total (*n* = 349)**	**GOSE score ≤ 4 (*n* = 233)**	**Number of patients with data**	**GOSE score ≥ 5 (*n* = 116)**	**Number of patients with data**	**p**
Location of other bodily injuries			233		116	
-Facial	140 (40.1)	90 (38.6)		50 (43.1)		0.422
-Chest	160 (45.8)	102 (43.8)		58 (50.0)		0.272
-Abdominal	86 (24.6)	50 (21.5)		36 (31.0)		0.051
-Extremity	136 (39.0)	80 (34.3)		56 (48.3)		0.012
-External	111 (31.8)	71 (30.5)		40 (34.5)		0.449
Abbreviated injury scale						
-Head	4 [3–5]	4 [4–5]		3 [2–4]		< 0.001
-Facial	2 [1–3]	2 [1–3]		1 [1–2]		0.192
-Chest	3 [1–3]	3 [2–3]		3 [1–4]		0.887
-Abdominal	2 [1–3]	1 [1–3]		2 [1–3]		0.940
-Extremity	2 [1–3]	2 [1–3]		2 [1–3]		0.683
-External	1 [1–2]	1 [1–2]		1 [1–1]		0.234
Number of associated injuries			233		116	0.336
None*	106 (30.4)	75 (32.2)		31 (26.7)		
1	64 (18.3)	48 (20.6)		16 (13.8)		
2	93 (26.6)	59 (25.3)		34 (29.3)		
3	18 (5.2)	11 (4.7)		7 (6.0)		
4	11 (3.2)	6 (2.6)		5 (4.3)		
5	57 (16.3)	34 (14.6)		23 (19.8)		

Data are expressed as the median (25-75^th^ percentile) and number (percentage). * Traumatic brain injury regardless of the AIS score for other injuries

**Table 3:** Intracranial pressure monitoring, intracranial hypertension and need for neurosurgery

		** ≥ 65 years**				
**Variables**	**Total (*n* = 349)**	**GOSE score ≤ 4 (*n* = 233)**	**Number of patients with data**	**GOSE score ≥ 5 (*n* = 116)**	**Number of patients with data**	**p**
Intracranial pressure monitoring	148 (63)	101 (68)	148	47 (54)	87	0.029
Intracranial pressure monitoring Glasgow Coma Scale score ≤ 8	107 (73)	81 (73)	111	26 (72)	36	0.930
Intracranial hypertension, > 20 mm Hg	52 (35)	39 (39)		13 (28)		0.194
-Osmotherapy	94 (27)	77 (33)	233	17 (15)	116	< 0.001
-Barbiturates	28 (8)	22 (9)	233	6 (5)	116	0.167
-Hyperventilation	10 (3)	8 (3)	233	2 (2)	116	0.506
Neurosurgery*	92 (26)	65 (28)	233	27 (23)	116	0.356
-Extradural or subdural haematoma	65 (19)	48 (21)	233	17 (15)	116	0.179
-Lobectomy	10 (3)	5 (2)	233	5 (4)	116	0.310
-Decompressive craniectomy	6 (2)	5 (2)	233	1 (1)	116	0.668

Data are expressed as number (percentage). *included ventricular derivation

Among the 349 patients, the GOSE score was < 5 and ≥ 5 in 233 (67%) and 116 (33%) patients, respectively. Patients who had an unfavourable outcome were significantly older, male, had a lower GCS score at baseline and were more severely ill regardless of the severity assessment modality (Table 1). They were more prone to receive intracranial pressure monitoring (unless complete data are lacking) and osmotherapy (Table 3). Clinical data from hospitalization in the ICU is provided in Table 4, and no difference was found between patients with a unfavourable vs. favourable outcome.

**Table 4:** Clinical data from patients hospitalized in the ICU

		** ≥ 65 years**				
**Variables**	**Total (*n* = 349)**	**GOS score ≤ 4 (*n* = 233)**	**Number of patients with data**	**GOS score ≥ 5 (*n* = 116)**	**Number of patients with data**	**p**
Infection	155 (44)	103 (44)	233	52 (45)	116	0.912
-Ventilator-associated pneumonia*	128 (37)	89 (38)	233	39 (34)	116	0.403
-Bacteraemia	27 (8)	19 (8)	233	8 (7)	116	0.679
-Urinary tract infection	28 (8)	17 (7)	233	11 (10)	116	0.479
-Meningitis	7 (2)	6 (3)	233	1 (1)	116	0.432
-Surgical site infection	4 (1)	2 (1)	233	2 (2)	116	0.603
-Central venous catheter infection	6 (2)	5 (2)	233	1 (1)	116	0.668
Mechanical ventilation	335 (96)	226 (97)	233	109 (94)	116	0.245
Length of mechanical ventilation, days	9 [4–17]	9 [4–18]	223	9 |4–16]	108	0.352
ARDS**	69 (20)	46 (20)	233	23 (20)	116	0.985
Tracheotomy	32 (9)	23 (10)	233	9 (8)	116	0.519
Vasopressor	263 (75)	182 (78)	233	81 (70)	116	0.091
Length of vasopressor use, days	3 [2–5]	3 [2–5]	180	3 [2–6]	81	0.576
Renal replacement therapy	7 (2)	6 (3)	233	1 (1)	116	0.432

Data are expressed as the median (25-75^th^ percentile) and number (percentage). *Twelve patients experienced a second episode of ventilator-associated pneumonia (GOS score < 4 and ≥ 4, *n* = 8 and *n* = 4, respectively). **ARDS: acute respiratory distress syndrome

Patients who had an unfavourable outcome had a shorter length of stay in the ICU, mortality in the ICU and mortality at day 90 were significantly higher, and withdrawal or withholding of life-sustaining therapies in the ICU was more frequent (Table 5).

**Table 5:** Outcomes

		** ≥ 65 years**				
**Variables**	**Total**	**GOS score ≤ 4 (*n* = 233)**	**Number of patients with data**	**GOS score ≥ 5 (*n* = 116)**	**Number of patients with data**	**p**
Length of ICU stay, days	12 [6–23]	11 [5–23]	227	14 [7–22]	113	0.153
ICU mortality	141 (40)	141 (61)	233	0 (-)	116	< 0.001
Withdrawal or withholding of life-sustaining therapies in the ICU	140 (40)	137 (59)	233	3 (3)	116	< 0.001
Mortality at day 90	157 (45)	157 (67)	233	0 (-)	116	< 0.001

Data are expressed as the median (25-75^th^ percentile) and number (percentage)

**Fig. 3:**
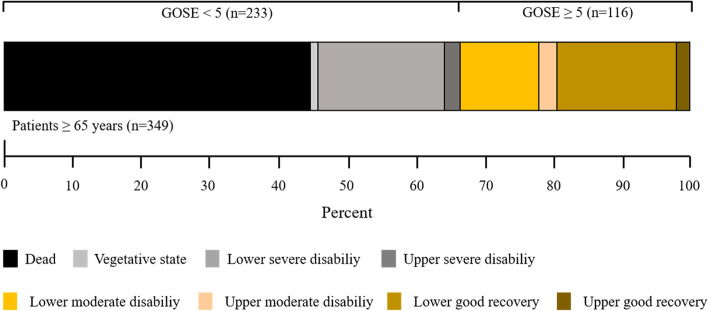
Distribution of glasgow outcome scale extended scores at the 3-month follow-up

The proportion of patients in each category of the GOSE is provided in Fig. 3. Of the patients who were admitted with an initial GCS score ≤ 8, 184 (77%) had a GOSE score < 5, and 157 (45%) had died at 3 months.

To perform multivariate analysis, the following variables were selected: age, sex, history of chronic obstructive pulmonary disease, GCS score at admission, pupils (one or two dilated and nonreactive), ISS, SAPS II, vasopressor use, neurosurgery for extradural or subdural haematoma and osmotherapy. Variables independently associated with unfavourable outcomes were age, male sex, ISS, GCS score at baseline and the use of osmotherapy (Model 1, Table 6). For age, GCS score at baseline and ISS, the best thresholds for sensitivity and specificity in predicting unfavourable outcome at 3 months were ≥ 77 years, ≤ 9 and ≥ 25, respectively (Model 2, Table 7).

**Table 6:** Multivariate analysis of unfavourable outcomes at 3 months: Model 1 (AUC = 0.79, 95% CI [0.74–0.84])

Variables	OR	95% CI	p
Age (per one year increase from ≥ 65 years)	1.09	1.04–1.14	< 0.001
Sex (ref = Female)	2.94	1.70–5.11	< 0.001
Baseline GCS score (per one point decrease from a GCS score 1)	1.20	1.13–1.29	< 0.001
Injury severity score (per one year increase)	1.04	1.02–1.06	< 0.001
Osmotherapy (ref = No)	2.42	1.26–4.65	0.008

*OR* Odds ratio, *95% CI 95%* 95% Confidence interval, *GCS* Glasgow Coma Scale

**Table 7:** Multivariate analysis for unfavourable outcome at 3 months for variables providing the best sensitivity and specificity: Model 2 (AUC = 0.79, 95% CI [0.74–0.84])

Variables	OR	95% CI	p
Age (≥ 77 *vs.* < 77)	3.31	1.86–5.91	< 0.001
Male sex	2.65	1.54–4.58	< 0.001
Baseline GCS score (≤ 9 *vs.* > 9)	5.42	3.07–9.60	< 0.001
Injury severity score (≥ 25 *vs*. < 25)	2.65	1.58–4.44	< 0.001
Osmotherapy (ref = No)	2.27	1.17–4.39	0.015

*OR* Odds ratio, *95% CI* 95% Confidence interval, *GCS* Glasgow Coma Scale

The rates of unfavourable outcomes in men and women were 72% and 56%, respectively, while comorbidities (except significantly more alcohol addiction in males) and severity of TBI assessed by the GCS score and ISS did not significantly differ (data not shown). The contribution of head injury into the ISS score was 69% (unfavourable 73% *vs*. favourable outcome 61%, *p* = 0.001).

## Discussion

In patients aged ≥ 65 years admitted to the ICU for TBI regardless of their initial GCS score, unfavourable functional outcomes assessed by a GOSE score < 5 were found in 67%, and only 7 patients (2%) fully recovered or had minor symptoms that did not affect daily life (GOSE score = 8). Factors associated with unfavourable outcomes were age, male sex, GCS score, ISS and use of osmotherapy during their course of disease. Although the proportion of surgical interventions other than neurosurgery was not collected in our study, the number of traumatic associated lesions and complications during ICU stay did not differ between patients with favourable *vs.* unfavourable outcomes, suggesting that the severity of TBI was the main cause of an unfavourable outcome.

It is well known that compared to younger patients, elderly patients who experience TBI have a poorer outcome [6, 19], but few studies have focused on older patients admitted to the ICU [4, 5, 20]. A meta-analysis reported 11 studies from 1986 to 2008 and outcomes in TBI patients ≥ 60 years old. Mortality in patients who had severe (GCS score ≤ 8; 10 studies), moderate (GCS score 9–12; 5 studies) and mild (GCS score 13–15; 6 studies) TBI was 79.3%, 42.5% and 10.7%, respectively, and unfavourable outcomes (GOS score 2–3) were 13.8%, 29.5% and 7.0%, respectively [21]. Nevertheless, this meta-analysis now included old studies (5 prior to 2000) with patients not always managed in the ICU, of various ages and with assessments of the outcome at various times. More specifically, in a cohort of 1366 patients from 3 neurosurgical ICUs in Italy, Stocchetti et al. showed that 6-month mortality and unfavourable outcomes (GOS score 1–3) increased with age, particularly for patients ≥ 60 years old [5]. In a prospective multicentre study, outcome at 1 year was studied in 100 TBI patients who had > 65 years and an initial GCS score of 3 or 4. ICU mortality was 76%, and at 6 months, only 9 patients had a favourable outcome (GOS score 1–3) [20]. Recently, a prospective registry-based cohort study performed in the state of Victoria, Australia, focused on outcomes at 6 months in patients ≥ 65 years admitted for TBI [4]. In the subgroup of 263 patients managed in the ICU, 12% were functional independent (GOSE score 5–8), 15% were dependent (GOSE score 2–4) and 73% had died at 6 months [4]. Our results are in agreement with these previous studies, emphasizing a high ICU and 3-month mortality and unfavourable functional outcome in TBI older patients. As reported by Maiden et al., we found that age was independently associated with unfavourable outcomes, and in our study, a threshold of ≥ 77 years indicated the worst outcome.

Cerebral injuries had a major impact on outcome, although the severity of associated injuries, assessed by the AIS, and the cumulative number of body injuries did not seem to affect outcome. Head AIS severity was previously associated with low odds of functional recovery in univariate analysis, and an ISS ≥ 25 was shown to be independently associated with an unfavourable outcome [4]. In our study, ISS was also an independent risk factor for an unfavourable outcome, and the best threshold was also ≥ 25. Interestingly, in our study, the contribution of the TBI to the ISS value was high, particularly in patients who had an unfavourable outcome, confirming the high value of the severity of head trauma on outcome. From this perspective, it is not surprising that we found that the initial GCS, reflecting the severity of TBI, was an independent risk factor for unfavourable outcomes, with a threshold ≤ 9 being the most relevant to predict unfavourable outcomes. Usually, a GCS score > 8 is considered moderate. Nevertheless, the impact of a TBI on outcome in elderly individuals may lead us to question the relevance of a higher initial GCS score threshold in this population. Thus, the presence of several comorbidities, psychiatric history and/or frailty may amplify the consequences of a TBI that would be considered less severe in a younger patient. In this context, it was shown that compared with that in a younger population, even a moderate head injury resulted in increased mortality in subjects over 64 years of age [9, 22]. Nevertheless, the initial GCS score alone did not allow good discrimination of the outcomes. Indeed, in our cohort, 47.5% of the patients who had a GCS score ≤ 8 also had a GOSE score ≥ 5 at 3 months.

We also found that male sex was independently associated with unfavourable outcomes. Head injuries are more frequent in men, but the proportion is balanced and/or reversed in older patients [1, 5]. However, the impact of sex on outcome provides various results [23]. In a review including 156 studies, the crude results reported a worse outcome in women, but analysis focused on moderate and severe TBIs (GCS score 3–12) showed a higher proportion of good outcomes in women compared to men [23]. Similarly, outcomes were better in women in large studies (> 10,000 patients) than in smaller studies [23]. The extent to which age may also affect the impact of sex on outcome also produces divergent results [24]. In analysing the sex-age interaction on outcome, comorbidities, degree of frailty, and severity of associated injuries need to be taken into account [24]. In our study the prevalence of unfavourable outcomes in men and women was 72.4% and 57.3% respectively while except for alcohol abuse comorbidities and severity of TBI did not differ.

Finally, the use of osmotherapy was found to be associated with unfavourable outcomes. The administration of osmotherapy probably reflects the detection of intracranial hypertension, but our study does not allow us to specify what motivated this prescription. Indeed, except for caricatured situations such as pupillary dilation, trans-cranial Doppler is a technique increasingly used in prehospital, emergency room and/or intensive care units to detect intracranial hypertension and can lead to the administration of osmotherapy without the use of an intracranial pressure sensor [25]. In our study the presence of intracranial hypertension measured by a pressure transducer did not differ between the two groups but we have too much missing data on the use of monitoring to make relevant conclusions.

Our study has some limitations that must be underlined. First, as with all prospective cohorts with retrospective analysis, some data were missing or incomplete. However, we excluded patients with missing data on the primary outcome and reported the number of missing data for all other variables. Second, all patients were managed in ICUs experienced in the care of severe trauma patients, including head trauma patients. Our results can only be applied to these patient populations and may be difficult to extrapolate to other care systems, although our results are in agreement with other studies performed in the same population. Third, the threshold of 65 years of age to define an elderly population may be debatable. However, given the unfavourable short- and long-term prognosis of severe trauma patients, particularly those suffering from head trauma, an age of 65 years is usually retained [4, 5]. Fourth, we collected data on the outcome at three months, and it cannot be excluded that an improvement can be observed beyond this date. Indeed, it was proposed that functional status should preferably be assessed from 6 months or beyond. It was also shown that the best improvement occurred between 2 weeks and 3 months, with a gain of approximately 1 point in the GOSE, and, to a lesser extent, between 6 and 12 months, with a gain of 0.2 points [26]. However in 263 TBI patients aged > 65 years the functional status did not significantly improve after 6 months [4]. Finally, in a recent multicentre study evaluating the effect of continuous hypertonic saline *vs.* standard care on outcome at 6 months, it appeared that the prevalence of a good neurologic outcome (GOSE score 6–8) did not significantly differ between 3 and 6 months (115/351 (32.8%) *vs.* 122/359 (34.0%)) [27]. Fourth, we did not evaluate frailty in our patients. Frailty is now recognized as an important determinant in outcome, notably in elderly ICU patients [28, 29]. A recent study evaluated a specific frailty score (CENTER-TBI frailty index) from a cohort of 2993 head injury patients. Among patients admitted to the ICU (1742 patients), the impact of frailty on adverse outcomes (GOSE score ≤ 4 at 6 months) was not as strong in the ICU as it was in a conventional hospital ward (cumulative OR [95% CI] = 1.02 [1.01–1.03] *vs*. 1.04 [1.03–1.06], *p* < 0.001), underlining the importance of the severity of head injury rather than frailty alone [29]. Fifth, the high frequency of withdrawal or withholding of life-sustaining therapies in ICU may have affected prognosis. In a prospective study performed in Europe and Israel, the majority of deaths in patients who experienced TBI was related to withdrawal of care (≈ 90% in western and ≈ 96% in northern Europe) [30]. In our study, it was difficult to draw clear explanations for these findings, as neither the reasons for withdrawal or withholding of life-sustaining therapies (WWLST) nor the timing of WWLST were collected, which may cause uncertainty when applied latter in the course of disease [30]. Nevertheless, the fact that few patients were in a vegetative state at 3 months may suggest that futility was the main reason for WWLST. Finally, according to the design of our study, we cannot exclude the possibility that other clinical and/or biological variables of potential interest were not collected.

## Conclusions

In TBI patients at least 65 years old admitted to intensive care, age ≥ 77 years, male sex, baseline GCS score ≤ 9, ISS ≥ 25 and use of osmotherapy were predictors of unfavourable outcome. These data may help physicians in the approach to determining the prognosis and providing information to relatives. In this population, the proportion of associated lesions does not seem to play a significant role in the outcome.

## Data Availability

The datasets used and/or analysed during the current study are available from the corresponding author on reasonable request.

## Abbreviations

TBI: Traumatic brain injury
ICU: Intensive care unit
GOSE: Glasgow Outcome Scale Extended
GCS: Glasgow Coma Scale
ISS: Injury Severity Score
SAPS II: Simplified Acute Physiology Score II
SOFA: Sequential Organ Failure Assessment
AIS: Abbreviated Injury Scale
ARDS: Acute Respiratory Distress Syndrome
AUC: Area Under the Curve
ROC: Receiver Operating Characteristic
